# Concurrent participation in breast, cervical, and colorectal cancer screening programmes in Denmark: A nationwide registry-based study

**DOI:** 10.1016/j.ypmed.2022.107405

**Published:** 2023-02

**Authors:** Sisse Helle Njor, Bo Søborg, Mette Tranberg, Matejka Rebolj

**Affiliations:** a University Research Clinic for Cancer Screening, Department of Public Health Programmes, Randers Regional Hospital, Randers, Denmark; b Department of Clinical Medicine, Aarhus University, Aarhus, Denmark; c Cancer Prevention Group, School of Cancer & Pharmaceutical Sciences, Faculty of Life Sciences & Medicine, King's College London, London, UK

**Keywords:** Mass screening, Early detection of cancer, Breast neoplasms, Uterine cervical neoplasms, Colorectal neoplasms

## Abstract

Women in Denmark are invited to breast, cervical, and colorectal cancer screening in their fifties and sixties. We determined the patterns of concurrent participation in the three programmes. Participation in organised cancer screening was determined using the highly complete Danish population and health care registers for all women aged 53–65 years on 31 March 2018 who continuously resided in Denmark since 1 April 2012. Data were linked using unique personal identification numbers. We studied overall and cancer-specific proportions of women undergoing screening for all three, two, one, and none of the cancers. Among all 468,507 women, 406,306 (87%) participated in breast, 345,768 (74%) in cervical, and 316,496 (68%) in colorectal cancer screening. Despite high participation, only 255,698 (55%) women were screened for all three cancers, while 123,469 (26%) were screened for two, 54,538 (12%) for one, and 34,802 (7%) were not screened for any cancer. Cancer-specific patterns were highly heterogeneous across the population but changed little after accounting for women's medical history. A significant proportion of women who are screened for a specific cancer remain unscreened for other cancers. The consistency of these data at the international level requires a reconsideration of invitational practices for organised screening.

## Highlights

• Participation in Danish breast, cervical, and colorectal cancer screening is >70%.
• Only 55% of the targeted women were screened for all three cancers.
• Most women screened for one or two cancers underwent breast cancer screening.

## 1 Introduction

In several countries, breast, cervical, and colorectal cancer screening has been offered through organised programmes. While screening can reduce cancer-specific mortality,(Laara et al., 1987; Njor et al., 2012; Zauber, 2015) these programmes require a high uptake to demonstrate effectiveness at the population level.(Weller and Campbell, 2009; Burger and Kim, 2014) Despite generally high levels of support for screening among the general population(Waller et al., 2015) and low financial barriers for participants, these programmes have achieved variable participation rates. In Denmark, 84% of the invited women participate in breast cancer screening, whereas 73% and 60% tend to be screened for cervical and colorectal cancer, respectively.(Styregruppen for DKLS, 2019; Dansk Kvalitetsdatabase for Mammografiscreening, 2021; Dansk Tarmkræftscreeningsdatabase, 2019) In other countries, where screening is also offered for free, similar or lower participation rates have been reported.(Integraal Kankercentrum Nederland, 2022; Eurostat, 2020; Integraal Kankercentrum Nederland, 2019; European Commission, 2017)

Furthermore, recent studies from England, Scotland, France, USA, and Japan have reported on the participation among the population recommended for screening for all three cancers, which usually includes women older than 50 years. These studies reported surprisingly low proportions of women who concurrently participated in screening for all three cancers, roughly between 10% and 50%, whereas the proportions of those who were screened for only one or at most two cancers varied between 30% and 50% and few tended to remain unscreened for all three cancers.(Ishii et al., 2021; McCowan et al., 2019; Rebolj et al., 2020; Smith et al., 2019; Dawidowicz et al., 2020) Most of these studies, however, used either small data sets, self-reported data, or were undertaken in settings where screening invitations are not sent systematically.

Using highly complete national registers, we aimed to determine the pattern of participation in cancer screening in Denmark, in women who are invited by all three population-based, organised programmes.

## 2 Methods

### 2.1 Cancer screening in Denmark

Since 2007, women aged 50–64 have been recommended for cervical cancer screening every five years, whereas younger women have been recommended for three-yearly screening. Invitations are sent only to women without a cervical test in the recommended age-appropriate interval. An appointment for a clinician sample is scheduled at primary care, often with several months of delay following the invitation and up to two reminders.(Styregruppen for DKLS, 2019) Women without a cervix are ceased from the programme.

Nationwide biennial breast cancer screening of women aged 50–69 years with mammography started in late 2007. Prior to that, population-based screening was only offered in some administrative units. Women receive an invitation with a pre-booked, yet changeable, appointment, and a reminder if they do not attend. They are recommended to re-join the programme after successful treatment of breast cancer.

Colorectal cancer screening using self-collection for faecal immunohistochemistry testing (FIT) was introduced in March 2014. To ensure sufficient colonoscopy capacity for FIT-positive individuals, the first invitation round was only planned to end on 31 December 2017. Consequently, the programme's first-round invitations were sent to all individuals aged 50–74 years at some point in 2014–2017.(Njor et al., 2018) Non-participants receive one reminder. Individuals undergoing surveillance after a colorectal cancer diagnosis are advised not to participate. Those with inflammatory bowel disease are advised to consult their treating physician before deciding to participate.

Screening tests and follow-up of abnormalities are free of charge.(Christiansen, 2002) All three programmes are routinely monitored for the uptake and various other quality indicators to identify any issues which require attention.(Styregruppen for DKLS, 2019; Dansk Kvalitetsdatabase for Mammografiscreening, 2021; Dansk Tarmkræftscreeningsdatabase, 2019)

### 2.2 Data sources

The Danish central population register was used to identify the study population.(Pedersen, 2011) The unique personal identification numbers issued to all residents upon birth or immigration are mandatory for registration in this and other registers, and were used for linkage.

Information on cervical cancer screening participation was obtained from the nationwide pathology database.(Bjerregaard and Larsen, 2011) This database includes cervical cytology and histology from private practices and hospitals and is used by the programme to identify women who are due an invitation. Consistent with the official statistical reports, we included all records describing cytological samples or human papillomavirus tests from the uterine cervix or vagina.(Styregruppen for DKLS, 2019)

Information on invitations and participation in colorectal and breast cancer screening was retrieved from the Danish colorectal(Thomsen et al., 2017) and breast(Mikkelsen et al., 2016) cancer screening databases. The colorectal database receives information directly from the administrative database in charge of the invitations, registration of participation, and of the follow-up of FIT-positive participants. The breast database obtains information on invitations from the relevant administrative databases, and on screening mammographies from the Danish national hospital registration.(Lynge et al., 2011) Hospital registration includes non-psychiatric in-patient, outpatient, and emergency admissions; it also attaches a specific code to screening mammographies.

We retrieved information on comorbidities, hysterectomies, and inflammatory bowel disease from the hospital registration. Information on cancer cases was retrieved from the pathology database.

All data were retrieved until 31 March 2018.

### 2.3 Statistical analysis

**Fig. 1:**
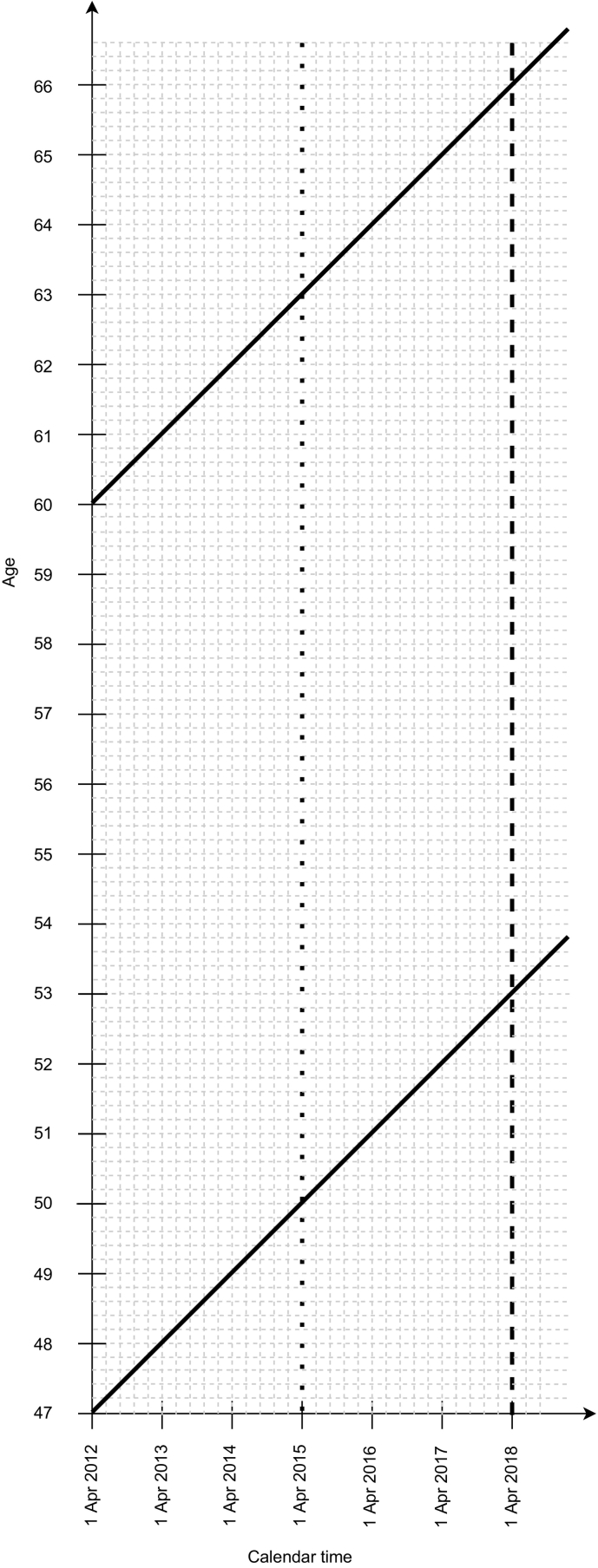
Lexis diagram for the study. Legend (see also Methods). Period of data availability: 1 April 2012 (study start date, delineated with a solid vertical line ending in an arrow) to 31 March 2018 (study end date, delineated with a long-dash vertical line). Cohorts included in the analyses: Delineated by solid bold diagonal lines. Definition of screening participation: In the main analysis, at least one screening test for any of the three cancers between 1 April 2012 and 31 March 2018; or, in a supplementary analysis, at least one cervical and colorectal cancer screening test between 1 April 2012 and 31 March 2018 and at least one breast cancer screening (mammography) test between 1 April 2015 (delineated with a dotted vertical line) and 31 March 2018. Age eligibility: 53–65 years on 31 March 2018. The lower age limit (53 years) was defined as follows. In some regions, women eligible for mammography screening registered at the same general practice are invited at the same time during the two-year interval. Women aged just under 50 will then be invited for mammography screening for the first time sometime before they turn 52; if, additionally, a screening round is extended due to e.g., local capacity issues, women could be invited for the first time only at the age of 52. To avoid including women at the lower end of the breast cancer screening target age range who could not participate because they had not yet been invited, we limited age eligibility for the study to those aged at least 53 years. The upper age limit (65 years) was defined as follows. Women are invited for the last cervical cancer screening round between the ages of 60 and 64 years. This means that all women aged 65 or younger should have been invited for cervical screening within the last 6 years, which was the definition of being up to date with cervical screening in our study (recommended as five years plus allowing one year for reasonable delays).

The study population were women aged 53–65 years on 31 March 2018, who lived in Denmark continuously since 1 April 2012 (Fig. 1).

For breast cancer, we counted women as having been screened if they had a screening mammography record between 1 April 2012 and 31 March 2018, following an invitation in the previous two years. For cervical cancer, women with at least one cervical screening test in the studied period were counted as participants. For most, this six-year period represented a single screening round, allowing for a delay to schedule the appointment of up to one year in line with the programme's annual statistical reporting.(Styregruppen for DKLS, 2019) For colorectal cancer, women with at least one FIT test by the end of March 2018, thus allowing for at least three months to return the completed test, were counted as participants.

We categorised women into groups of those who participated either in three, two, one, or none of the programmes. For women participating in a specific programme, we calculated the relative proportion (RP) participating in additional programmes. The 95% confidence intervals (CI) for the RPs were calculated assuming that their logarithms were approximately normally distributed.

We undertook supplementary analyses to assess the robustness of our findings. During the studied period, women could receive up to three invitations for breast cancer screening. We repeated the analysis by including only mammographies from 1 April 2015 onwards. Finally, we determined whether screening non-participation could be explained because certain groups of women had been at least temporarily advised not to participate. This included women with a diagnosis of colorectal or cervical cancer, hysterectomy, and inflammatory bowel disease (where the relevant data were retrieved from the beginning of the national hospital registration in 1977 until 31 March 2012), or because they had severe comorbidities i.e., those with a Charlson comorbidity index score(Charlson et al., 1987) ≥3 (where the relevant data for were retrieved for the 20-year period between 1 April 1992 and 31 March 2012).

### 2.4 Ethics approval and consent to participate

According to EU General Data Protection Regulation (article 30), the project was listed at the record of processing activities for research projects in Central Denmark Region (journal number: 1–16–02-90-17). According to Danish legislation, notification of register-based research projects to the research ethics committee is not required. Therefore, this study may be conducted without an approval from ethics committees.

## 3 Results

**Fig. 2:**
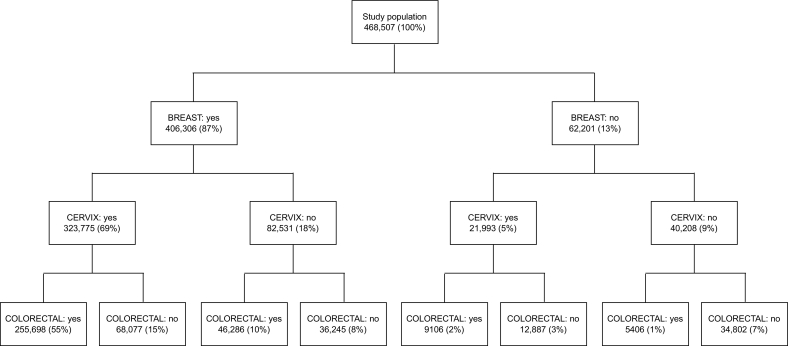
Breakdown of the study population.

In total, 468,507 women satisfied the inclusion criteria (Fig. 2). Of these, 406,306 (87%) underwent breast, 345,768 (74%) underwent cervical, and 316,496 (68%) underwent colorectal cancer screening (Table 1). Furthermore, 255,698 (55%) were screened for all three cancers, 123,469 (26%) were screened for two, 54,538 (12%) were screened for one, and 34,802 (7%) were screened for none, with very similar patterns across the age span despite the decreasing participation in cervical cancer screening and the slightly increasing participation in colorectal cancer screening (Table 2). Fig. 2 further shows that all possible combinations of participation in the three programmes were represented in this population, even if some patterns were rare such as participation only in colorectal cancer screening (1%). Another interesting pattern was that among 178,007 (38%) women who were screened for one or two cancers, a majority, 150,608 (85%, or 32% of the study population), underwent breast cancer screening.

**Table 1:** Conditional probabilities for participation in the three cancer screening programmes.

Participation in screening for cancer	If participated in screening for cancer			
	Breast	Cervical	Colorectal	None
Breast	X	323,775/345,768 94%	301,984/316,496 95%	36,245/71,047 51%
Cervical	323,775/406,306 80%	X	264,804/316,496 84%	12,887/47,689 27%
Colorectal	301,984/406,306 74%	264,804/345,768 77%	X	5406/40,208 13%
None	36,245/406,306 9%	12,887/345,768 4%	5406/316,496 2%	X
Total	406,306/468,507 87%	345,768/468,507 74%	316,496/468,507 68%	34,802/468,507 7%

Note. Proportions were rounded to the nearest integer.

**Table 2:** Participation in the three cancer screening programmes by age group.

Age group (years)	Participation in cancer screening						
	Breast	Cervical	Colorectal	All three	At most two	At most one	None
53–55	87%	78%	66%	55%	27%	12%	7%
56–60	87%	72%	68%	54%	27%	12%	7%
61–65	86%	71%	69%	54%	26%	11%	8%
Total	87%	74%	68%	55%	26%	12%	7%

Note. Proportions were rounded to the nearest integer.

Participation in one screening programme was associated with increased participation in other programmes (Table 1, Table 3). Only small proportions of women who participated in any of the programmes did not obtain any further screening: 9% (36,245/406,306) among those who participated in breast, 4% (12,887/345,768) among those who participated in cervical, and 2% (5406/316,496) among those who participated in colorectal cancer screening (Table 1). Nevertheless, only 255,698 (63%) of 406,306 women who underwent screening for breast cancer also participated in both other programmes (Table 3). Among 62,201 women who did not undergo screening for breast cancer, 9106 (15%) underwent both cervical and colorectal screening and 34,802 (56%) remained completely unscreened. Similar patterns were observed for cervical and colorectal cancer screening. In general, >90% of women obtained screening for breast cancer if they had also been screened for cervical or colorectal cancers; >80% were screened for cervical cancer if they had also been screened for breast or colorectal cancers; and >70% were screened for colorectal cancer if they had also been screened for breast or cervical cancers (Table 1).

**Table 3:** Participation in breast, cervical, and colorectal cancer screening programmes, by whether a woman participated in a specific programme (*N* = 468,507).

Cancer screening participation	N	Participated in both other programmes		Participated in no other programme	
		N	RP (95% CI)	N	RP (95% CI)
Breast: No	62,201	9106	1 (ref)	34,802	1 (ref)
Breast: Yes	406,306	255,698	4.30 (4.22–4.38)	36,245	0.16 (0.16–0.16)
Cervical: No	122,739	46,286	1 (ref)	34,802	1 (ref)
Cervical: Yes	345,768	255,698	1.96 (1.95–1.98)	12,887	0.13 (0.13–0.13)
Colorectal: No	152,011	68,077	1 (ref)	34,802	1 (ref)
Colorectal: Yes	316,496	255,698	1.80 (1.79–1.81)	5406	0.07 (0.07–0.08)

Abbreviations. CI: confidence interval. RP: relative proportion.

**Fig. 3:**
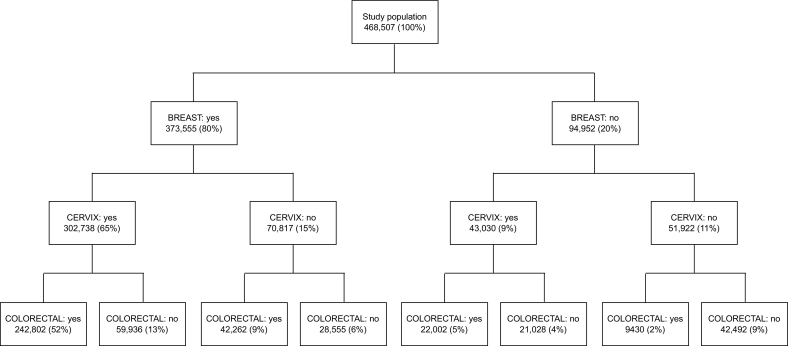
Breakdown of the total study population disregarding breast cancer screening participation before 1 April 2015.

Excluding around 8% women who had a previous hysterectomy increased the observed participation in cervical screening from 74% to 80% (not tabulated), which in turn increased the proportion of women who were screened for all three cancers from 55% to 60% (Supplementary Information, Fig. S1). Other exclusions, which affected only around 5% of the studied population, did not substantially affect participation at the population level. Including only screening mammographies in the last three years decreased the proportion of those considered as participating in breast cancer screening from 87% to 80% (Fig. 3). This decreased the proportion of women who were screened for all three cancers from 55% to 52%.

## 4 Discussion

### 4.1 Main findings

In Denmark, where women in their fifties and sixties are offered screening for early detection of cervical, breast, and colorectal cancers, only 55% were screened with all the recommended tests. A further 38% were screened for only one or at most two cancers, most often for breast cancer. Overall, we observed very heterogeneous participation patterns across the entire population, with all possible combinations of attended programmes represented in the data. Only a very small part of the observed patterns could be explained by women not attending cervical screening due to prior hysterectomies, whereas other factors such as severe comorbidities, or undergoing surveillance after cancer treatment had a limited effect when measured at the population level.

### 4.2 Strengths and weaknesses

The major strengths of this study were the inclusion of the entire female Danish population targeted for screening, and the fact that all information was retrieved from highly complete national administrative registers. This minimised the risk of selection and recall biases and allowed us to provide reliable estimates of the population's participation patterns.

Certain limitations ought to be acknowledged. Non-participation could to some extent be explained by women having actively opted out of receiving screening invitations. Their numbers are not reported in the programmes' official statistical reports,(Styregruppen for DKLS, 2019; Dansk Kvalitetsdatabase for Mammografiscreening, 2021; Dansk Tarmkræftscreeningsdatabase, 2019) but are small.(Harder et al., 2018) Danish registers do not offer complete information on bilateral mastectomies, which would be a reason to cease a woman from breast cancer screening. The incidence of bilateral mastectomy in Danish women is probably comparable to that reported for the UK,(Neuburger et al., 2013) which is low. Finally, we used the Charlson comorbidity index score calculated for the 20 years prior to the start of our study. Calculating the index based on an alternative version developed by the Royal College of Surgeons(Armitage and van der Meulen, 2010) resulted in fewer women being excluded from the analysis but did not otherwise change our results (not tabulated). The results also did not meaningfully change when we calculated the index based on diagnoses from the beginning of the registration in the national hospital register (since 1977, except for outpatient and emergency admissions since 1995) until the end of the follow-up in 2018 (not tabulated).

### 4.3 Comparison with the literature and implications for clinical practice

Our results are consistent with those reported previously, showing that while few women remained unscreened concurrently for all three cancers, a significant proportion did not participate in all three programmes.(Ishii et al., 2021; McCowan et al., 2019; Rebolj et al., 2020; Smith et al., 2019; Dawidowicz et al., 2020) They were also consistent with studies assessing correlations in participation for two types of cancer screening at a time, which reported that women who underwent screening for one cancer were more likely to participate in screening for other cancers.(Aklimunnessa et al., 2006; Wirth et al., 2014; Bertaut et al., 2018; Venturelli et al., 2019)

Non-participation in all or some of the recommended cancer screening has been observed in women from all sociodemographic backgrounds. Nevertheless, it appeared to be somewhat more frequent among women with lower attained education and those living in more deprived areas.(Ishii et al., 2021; Rebolj et al., 2020; Smith et al., 2019; Dawidowicz et al., 2020; Venturelli et al., 2019) In the USA, concurrent non-participation in screening for the three cancers was also found more frequently among women from certain racial backgrounds.(Smith et al., 2019) Some of the studies reported an association with women's smoking and other health-related behaviours,(Ishii et al., 2021; Smith et al., 2019; Venturelli et al., 2019) and with self-reported health.(Ishii et al., 2021; Venturelli et al., 2019) In our study, health-related issues such as comorbidities and hysterectomies appeared to explain non-participation only to a minor extent. In reality, the relationships between women's circumstances and screening participation are very complex and have been shown to vary by cancer type.(Ishii et al., 2021; Kotzur et al., 2020) This emphasises the need for in-depth studies(Kotzur et al., 2020) to more reliably disentangle causal pathways directly affecting the decisions regarding screening participation, and help develop robust policies.

This consistency of findings across the different screening settings and women's sociodemographic backgrounds requires a reconsideration of strategies with which women are informed about, and invited to, the different screening programmes. Various strategies have been proposed to improve screening participation in individual programmes. These have included e.g., offering self-sampling kits for human papillomavirus testing to non-attenders in cervical screening,(Yeh et al., 2019) second timed appointments for mammography,(Allgood et al., 2017) or changing the more cumbersome faecal occult blood tests, if used, with less cumbersome FIT tests.(Moss et al., 2017) Nevertheless, none of these approaches have increased screening participation by more than a few per-cent.

Data like ours now seem to suggest that the responsibility to invite women to cancer screening should perhaps be shared between the individual programmes. The three cancer screening programmes tend to be managed independently of one another. Women receive separate invitations and there tends to be little to no exchange of the information. In our study, almost all women who participated in one or two programmes were screened for breast cancer and these women represented 32% of the study population. This raises a question whether a mammography appointment could be used as a de facto additional reminder for women who are not up to date with cervical or colorectal screening, or both. Such a strategy would have to rely on communicating the women's screening participation status between the programmes and could work by providing advice and additional FIT and human papillomavirus self-sampling kits. In countries such as Denmark, this should be technically feasible as screening records are entered into administrative registers in real-time. The feasibility of this approach in routine mammography screening is being studied in an on-going Danish randomised controlled trial (http://clinicaltrials.gov: NCT05022511).(Helgestad et al., 2022) One of the issues that would require special consideration for implementation is an increased demand on the time of the screening team, adding to the already tight time constraints. Another issue is whether women would find this approach acceptable. This is also studied in the on-going Danish trial, but previous studies have shown that while most women found it acceptable to receive advice on other types of screening as they are attending for breast cancer screening, around 10% indicated that it may deter them from participating altogether.(Scott et al., 2021; Stevens et al., 2019) Nevertheless, studies in cervical cancer screening showed that significant proportions of under-screened women find it acceptable to be offered a self-sampling kit when they consulted primary care for unrelated reasons.(Lim et al., 2017; Peeters et al., 2020)

## 5 Conclusion

Around half of Danish women in their fifties and sixties participated in all three cancer screening programmes, whereas around one in three participated in only one or two. The reasons for these heterogeneous patters require additional study but the current observations call for a consideration of new approaches to cancer screening invitations.

## Authors' contributions

Conteptualization: SHN, MR.

Data curation: BS.

Formal analysis: SHN, MR.

Methodology: SHN, MR.

Writing - original draft: SHN, MT, MR.

Writing - review and editing: SHN, BS, MT, MR.

## Funding information

**SHN**: The Health Research Fund of the Central Denmark Region (no. A1518).

**BS**: none declared.

**MT**: 10.13039/501100004836Independent Research Fund Denmark (grant no: 1057-00018B).

**MR**: 10.13039/501100000289Cancer Research UK (reference: C8162/A27047).

The funders had no role in designing the study; in the collection and analysis of the data; the writing of the manuscript; and the decision to submit.

## Declaration of Competing Interest

The authors declare the following financial interests/personal relationships which may be considered as potential competing interests.

**SHN**: received a speaking fee from Norgine.

**BS**: none declared.

**MT**: is undertaking studies using HPV test kits and CINtec plus kits sponsored by 10.13039/100004337Roche, outside of the submitted work.

**MR**: received funding from 10.13039/501100002141Public Health England for epidemiological analyses of various clinical study datasets; member of various expert committees advising the English Cervical Screening Programme; attended meetings with various human papillomavirus assay manufacturers; fee for lecture in the last four years from Hologic, paid to employer.

## Data Availability

The data that support the findings of this study are available from The Danish Health Data Authority and The Danish Clinical Quality Program – National Clinical Registries (RKKP). Restrictions apply to the availability of these data, which were used under license for this study. Data may be available upon reasonable request to The Danish Health Data Authority and The Danish Clinical Quality Program – National Clinical Registries (RKKP).

## Appendix A Supplementary data

Supplementary material Image 1
