# Supplementary material for: Concurrent participation in breast, cervical, and colorectal cancer screening programmes in Denmark: A nationwide registry-based study
Source: Prev Med. 2023 Feb;167:107405. doi: 10.1016/j.ypmed.2022.107405 (PMC10265133; doi:10.1016/j.ypmed.2022.107405)
Supplement: Supplementary file 1 — Supplementary material [file mmc1.docx]

Department of Public Health Programmes

Randers Regional Hospital

13 December 2022

**Concurrent participation in breast, cervical, and colorectal cancer screening programmes in Denmark:**

**a nationwide registry-based study**

**SUPPLEMENTARY INFORMATION**

Sisse Helle Njor, Bo Søborg, Mette Tranberg, Matejka Rebolj

Content: additional breakdowns of the data for supplementary analyses.

Figure S1. Breakdown of the study population after exclusion of women with previous breast cancer (N=3342), hysterectomy (N=38,398), irritable bowel syndrome (N=8008), or a Charlson comorbidity score 3 or higher (N=9812).
